# Viral infections and invasive group a streptococcal disease incidence during 2022–2023

**DOI:** 10.1016/j.lanepe.2025.101581

**Published:** 2026-01-08

**Authors:** Rosalie Lear, Shiranee Sriskandan, Tom Parks

**Affiliations:** Department of Infectious Disease, Imperial College London, UK

Lenglart and colleagues reported an association between viral infections and invasive *Streptococcus pyogenes* disease (also known as invasive group A streptococcal disease; iGAS).^1^ While this issue has caused considerable concern, we agree that causality cannot be established from ecological correlations and wish to highlight the impact of surveillance heterogeneity and residual confounding.

First, iGAS definitions differed across the countries in the study. In the UK, laboratory isolation, including molecular tests but not antigen testing, is used for confirmation, whereas criteria elsewhere vary, complicating cross-country comparisons. Use of Google Trends to infer viral activity is also problematic. The studies cited assessed single viruses and not several concurrently; none examined varicella-zoster virus trends using this method; and using the term “bronchiolitis” as a substitute for respiratory syncytial virus (RSV) is probably unreliable beyond infancy.

Second, confounders were not considered in detail. Recent work by Dokal and colleagues^2^ demonstrated that post-pandemic “immune debt” was an important factor in the rise of paediatric iGAS during 2022–2023, underscoring the need to adjust for altered population immunity in any analysis since population level changes in immunity will correlate with viral and bacterial rates.

Finally, correlation does not imply causation. UK data shows paediatric iGAS cases were increasing in April to June 2022, preceding major viral resurgences later that year (Fig. 1). Individual-level data from large multinational cohorts are needed to address this nuance. Respiratory viral infections may well have played a role in the severity of iGAS but it is unlikely these caused the upsurge.

**Fig. 1:**
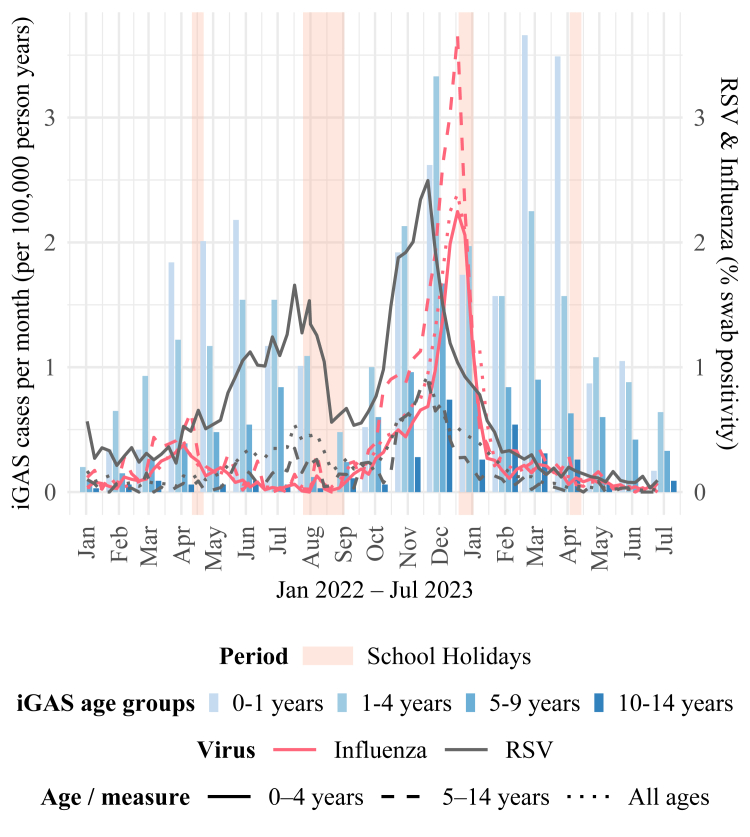
**Monthly iGAS incidence rate and weekly RSV and influenza swab positivity in England during 2022**–**2023.** Data were obtained from the UKHSA data portal and national respiratory virus reporting.^3^, ^4^, ^5^, ^6^ Dates of school holidays in England were obtained from the London Borough of Waltham Forest.

## Contributors

R.L. and T.P. had the idea for the manuscript. R.L. generated the first draft. T.P. and S.S. critically revised the manuscript. All authors approved the final version.

## Declaration of interests

R.L. reported a research grant from the Medical Research Council. S.S and T.P reported research grants from the Wellcome Trust, Leducq Foundation and Medical Research Council.
